# Integrative taxonomy confirms three species of *Coniocarpon* (Arthoniaceae) in Norway

**DOI:** 10.3897/mycokeys.62.48480

**Published:** 2020-01-23

**Authors:** Andreas Frisch, Victoria Stornes Moen, Martin Grube, Mika Bendiksby

**Affiliations:** 1 NTNU University Museum, Norwegian University of Science and Technology, 7491 Trondheim, Norway; 2 Institute of Plant Sciences, Karl-Franzens-University Graz, Holteigasse 6, 8010 Graz, Austria

**Keywords:** Arthoniales, Bayesian inference, maximum likelihood, morphology, mtSSU, nucITS, phylogeny, *RPB2*

## Abstract

We have studied the highly oceanic genus *Coniocarpon* in Norway. Our aim has been to delimit species of *Coniocarpon* in Norway based on an integrative taxonomic approach. The material studied comprises 120 specimens of *Coniocarpon*, obtained through recent collecting efforts (2017 and 2018) or received from major fungaria in Denmark, Finland, Norway and Sweden, as well as from private collectors. We have assessed (1) species delimitations and relationships based on Bayesian and maximum likelihood phylogenetic analyses of three genetic markers (mtSSU, nucITS and *RPB2*), (2) morphology and anatomy using standard light microscopy, and (3) secondary lichen chemistry using high-performance thin-layer chromatography. The results show three genetically distinct lineages of *Coniocarpon*, representing *C. cinnabarinum*, *C. fallax* and *C. cuspidans* comb. nov. The latter was originally described as Arthonia cinnabarina f. cuspidans and is herein raised to species level. All three species are supported by morphological, anatomical and chemical data.

## Introduction

Species recognition is crucial for improved natural resource management and biodiversity conservation (Lumbsch and Leavitt 2011). Species recognizing can be challenging in lichenized fungi due to unclear species boundaries and/or cryptic diversity (e.g., Leavitt et al. 2011; Molina et al. 2011; Carlsen et al. 2012; Lücking et al. 2014; Bendiksby et al. 2015). One such example is *Coniocarpon* DC.

*Coniocarpon* belongs in the family Arthoniaceae Rchb. of the order Arthoniales. The Arthoniales is one of the largest orders of predominantly lichenized crustose and fruticose taxa (Sundin and Tehler 1998), with around 1500 lichenized, lichenicolous and saprotrophic fungi (Frisch et al. 2014; Van den Broeck et al. 2018). About 800 species belong to the family Arthoniaceae (Frisch and Thor 2010). The largest genus of the family is *Arthonia* Ach., with around 500 species (Grube 1995). As currently circumscribed, *Arthonia* is a polyphyletic genus with high diversity in morphology, chemistry, ecology, distribution and habitat preferences (Frisch et al. 2015). In the process of splitting *Arthonia* into monophyletic taxa, several genera have been described, emended or resurrected recently, of which *Coniocarpon* DC. is one (Frisch et al. 2014). Others include *Bryostigma* Poelt and Döbbeler, *Coniarthonia* Grube, *Felipes* (Ach.) Frisch and G.Thor, *Inoderma* (Ach.) Gray, *Melarthonis* Frisch and G.Thor, *Pachnolepia* A.Massal. and *Synarthonia* Müll.Arg. (Grube 2001; Frisch et al. 2014, 2015; Van den Broeck et al. 2018).

*Coniocarpon* is a small genus of four accepted species that are mainly distributed in humid tropical to warm-temperate regions of the world (Aptroot and Sparrius 2003; Coppins and Aptroot 2009; Van den Broeck et al. 2018), reaching higher latitudes, for instance, in the boreo-nemoral rainforests and other highly oceanic habitats in Norway (Blom et al. 2015). The genus is characterized within Arthoniaceae by crystalline orange, red and purple quinoid pigments in the ascomata that are dissolving with purple solution in K, by hyaline, macrocephalic, transversely septate ascospores that turn brownish with granular ornamentation in the epispore at late maturity, and by rounded to lirellate ascomata (Frisch et al. 2014, 2018; Van den Broeck et al. 2018). The widespread species *C. cinnabarinum* DC. and *C. fallax* (Ach.) Grube show marked morphological differentiation at the world level indicative for polyphyletic taxa.

The brightly pigmented ascomata in combination with their high value for nature conservation are two reasons why *C. cinnabarinum* and *C. fallax* are frequently collected in Norway. Both species are listed as vulnerable (VU) on the *Norwegian Red List of Species 2015* (as *A. cinnabarina* and *A. elegans*; Henriksen and Hilmo 2015). The boreo-nemoral rainforests, located between 58–62°N (DellaSala et al. 2011), are also categorized as VU in the *Norwegian Red List of Nature Types 2018* (Blom 2018). *Coniocarpon* and other taxa restricted to these forests are of particular importance for biodiversity conservation due to their limited distribution, which makes them vulnerable to extinction at a regional level.

Based on preliminary morphological and molecular evidence, the presence of three distinct taxa of *Coniocarpon* in Norway is hypothesized. This study aims to test this hypothesis by delimiting the taxa of *Coniocarpon* in Norway using an integrative taxonomic approach, including molecular, morphological and chemical data. All available specimens of *Coniocarpon* housed in fungaria in Denmark, Norway and Sweden have been revised. Species descriptions and an identification key are provided for all taxa and their distributions in Scandinavia are mapped.

## Materials and methods

### Collection methods and handling of fresh specimens

New specimens of *Coniocarpon* for this study were collected in boreo-nemoral rainforests on the west coast of Norway from Vest-Agder to Møre og Romsdal in 2017 and 2018. Specimens were placed in paper bags, allowed to air-dry and later stored at -20 °C due to prior knowledge of fast DNA degradation (e.g., Frisch et al. 2014). After DNA extraction, specimens were incorporated for long-term storage and access in the fungarium in Trondheim (TRH). Additional specimens were made available from fungaria in Bergen (BG), London (BM), Copenhagen (C), Edinburgh (E), Helsinki (H), Oslo (O), Paris (PC), Prag (PRA), Stockholm (S), Trondheim (TRH) and Uppsala (UPS).

### Taxon sampling

In total, 26 specimens were used for the molecular data production; 25 specimens from Norway and 1 specimen from Great Britain. Outgroup taxa and additional sequences of *Coniocarpon* were downloaded from GenBank, in total 32 sequences. Eighteen of these (nine mtSSU and nine *RPB2*) represent the nine outgroup taxa, whereas 14 (eight mtSSU and six *RPB2*) were from eight specimens of *Coniocarpon* originating from Great Britain, Japan, Norway, Rwanda and Uganda. Outgroup taxa were selected based on their phylogenetic position in Frisch et al. (2014) and Van den Broeck et al. (2018).

### DNA extraction and sequencing

DNA was isolated from specimens up to one year old. Genomic DNA was extracted following one of three methods. (1) Five to eight ascomata were sampled in 2 ml microcentrifuge tubes with two 3 mm diam. tungsten carbide beads each and crushed into a fine powder using a Retsch TissueLyser II. Subsequently, genomic DNA was extracted using the E.Z.N.A. SP Plant DNA Kit (Omega BIO-TEK, USA) following the manufacturer’s instructions. (2) Three to five ascomata were sampled directly in 0.2 ml Eppendorf PCR Tubes with 30 µl Dilution Buffer (Phire Plant Direct PCR Kit, ThermoFisher Scientific, Lithuania) and crushed with tweezers. (3) Small cuttings (ca. 50–100 µm × 50–100 µm in size) of the hymenium were sampled in 0.2 ml Eppendorf PCR Tubes and directly used for PCR amplification. The Phire Plant Direct PCR Kit (ThermoFisher Scientific, Lithuania) was used for PCR amplification in all three methods. Each PCR reaction contained 10 µl 2× Phire Plant PCR Buffer, 0.4 µl Phire Hot Start II DNA Polymerase, 1 µl of each primer for all genetic markers except *RPB2* (1.5 µl of each primer), 1 µl genomic DNA (1:1) or the lichen sample, and was filled with H_2_O to the final volume of 20 µl. If PCR amplification resulted in weak products, 2 µl genomic DNA (1:10) was added. PCR amplification was done for the mtSSU, nucITS and the protein-coding gene *RPB2* with the following primers: mtSSU1 + mtSSU3R (Zoller et al. 1999), ITS-1F + ITS4 (Larena et al. 1999, White et al. 1990) and *RPB2*-7cF + *RPB2*-11aR (Liu et al. 1999), respectively. PCR cycling conditions for mtSSU and nucITS started with an initial denaturation at 98 °C for 5 min, followed by 40 cycles of 98 °C for 5 s, 59 °C for 5 s, and 72 °C for 30 s, followed by a final extension of 72 °C for 1 min. For *RPB2*, annealing temperature was set to 57 °C. The PCR products were visualized on a 1% agarose gel stained with SYBR Safe DNA gel stain (ThermoFisher Scientific, USA) under UV light. Clean PCR products (i.e., those lacking visible contamination) were purified by adding 5 µl ExoSAP-IT Express PCR Cleanup (1:3 concentration; ThermoFisher Scientific, United Kingdom) to the PCR reactions. PCR reactions resulting in more than a single product were purified using the E.Z.N.A. Gel Extraction Kit (Omega BIO-TEK, USA) following the manufacturer’s instructions, except that we performed an additional wash buffer step. The PCR products were sent to Eurofins Genomics (Germany) for Sanger sequencing using the same primers as for the PCR reactions.

### Sequence alignment and phylogenetic analyses

The sequences were edited and aligned using BioEdit v.7.0.5.3 (Hall 1999). The identity of the sequences was verified using the nucleotide BLAST search in GenBank. For the examination of topological incongruence among gene trees, maximum likelihood (ML) bootstrapping analyses was carried out on each of the data sets using the RAxML-HPC Blackbox ver. 8.2.10 (Stamatakis 2014). The standard settings without estimation of the proportion of invariable sites (GTRGAMMA) were used. Topological incongruence was assumed if conflicting tree topologies were supported by ≥ 70% bootstrap support. Since topological incongruence could not be observed, maximum likelihood (ML) bootstrapping analysis was carried out on the concatenated three-locus dataset of 43 accessions for *Coniocarpon* using default settings and adding single genes as parameter partition. The RAxML analysis was stopped automatically after 402 bootstrap replicates using the MRE-based bootstopping criterion (Pattengale et al. 2010).

The best-fit evolutionary model for each partition using the Bayesian information criterion (BIC; Schwarz 1979) was estimated using PartitionFinder2 ver. 2.1.1. (Lanfear et al. 2016). The input data included both ingroup and outgroup taxa. The pre-set partitions were mtSSU, *RPB2*/1^st^ codon position, *RPB2*/2^nd^ codon position, *RPB2*/3^rd^ codon position, ITS1, 5.8S and ITS2. The Bayesian analysis was performed using MrBayes v.3.1.2 (Huelsenbeck et al. 2001; Ronquist and Huelsenbeck 2003). The MCMC run was using four independent chains and 10 million generations, sampling trees every 1000^th^ generation. After removal of a burnin of 25%, 7500 trees were summarized in a final Bayesian 50% majority-rule consensus tree. All phylogenetic analyses were run on the CIPRES Science Gateway (Miller et al. 2010). Phylogenetic trees were visualized using FigTree ver. 1.4.4 (Rambaut 2018). Informative characters were estimated for ingroup taxa per locus using Winclada ver. 1.61 (Nixon 1999–2002).

### Morphological and chemical investigations

The morphology of 120 specimens of *Coniocarpon* (Norway 87, Sweden 8, Denmark 16, Great Britain 7, Austria 1, Turkey 1) was studied. The morphology of all specimens was examined using a Leica M80 stereomicroscope and a Zeiss Standard Binocular microscope. Macroscopic photographs were taken with a Leica MZ16A stereomicroscope fitted with a Leica DFC420 camera. Microscopic photographs were taken with a Leica CTR6000 microscope fitted with a Leica DFC365 camera. Sections of ascomata and lichen thalli were cut by hand and mounted in water or lactic acid cotton blue (LCB). Length and width were measured for single ascomata as well as for aggregations composed of several ascomata. For the epithecium, exciple, hymenium and hypothecium, measurements were performed in LCB. Measurements of asci and ascospores were performed in water using squashed preparations. Only fully developed ascospores and commonly asci containing mature ascospores (sometimes asci without mature ascospores) were measured. Ascospore measurements are presented as (min.–)mean ± SD(–max.). The amyloid reaction of the apothecia was tested using 0.2% (Iodine _diluted_) and 1% (I), and 1% (I) solution after pretreatment with 10% potassium hydroxide (KOH) in water (KI). The quinoid pigments and Ca-oxalate crystals were measured in water and their shape studied. Later, the crystals reaction with KOH was observed. The quinoid pigments were identified by HPTLC (Arup et al. 1993) in solvent C. Quionid pigments are named according to Frisch et al. (2018) except for the newly identified A4.

### Distribution maps

Based on occurrence information of all revised specimens, the distribution of *Coniocarpon* species in Scandinavia was illustrated by adding a delimited text layer to a Wikimedia map from QuickMap services in QGIS ver. 3.6.2. (QGIS Development Team 2019). The younger specimens (i.e., collected from the mid-80s and onwards) were placed on the maps by their geographical coordinates, while older specimens (collected 1870–1983) lacking geographical coordinates were placed on the maps based on locality information. The Earth Point coordinate converter (http://www.earthpoint.us/Convert.aspx) was used to convert coordinates.

## Results

### Molecular data

A total of 75 new sequences were obtained from the 26 included specimens of *Coniocarpon* (mtSSU 26, nucITS 25, *RPB2* 24; Table 1). The lengths of the alignments (all accessions included) and the phylogenetically informative characters for the ingroup taxa were for mtSSU 889/61, ITS1 569/160, 5.8S 129/0, ITS2 239/84 and *RPB2* 867/127.

The selected substitution models for the five subsets in PartitionFinder2 were: 1) GTR+G for mtSSU, 2) K80+1 for *RPB2*/1^st^, 3) F81+I for 5.8S and *RPB2*/2^nd^, 4) HKY+G for *RPB2*/3^rd^ and 5) HKY+I for ITS1 and ITS2. The three gene-trees were congruent and a three-locus, concatenated dataset of 43 accessions were analyzed phylogenetically.

**Table 1.** Vouchers and their GenBank accession numbers. New sequences are indicated in bold. An en dash indicates missing data.

Specimens	Voucher	Country	mtSSU	nucITS	*RPB2*
*Arthonia didyma*	*Ertz 7587* (BR)	Belgium	EU704047	–	EU704010
*Arthonia granithophlia*	*Frisch 10/Se74* (UPS)	Sweden	KJ850981	–	KJ851107
*Arthonia physcidiicola*	*Frisch 11/Ug318* (UPS)	Uganda	KF707646	–	KF707657
*Coniocarpon cinnabarinum* 1	*Frisch* (TRH-L-29009)	Norway	**MN733983**	**MN734118**	**MN719396**
*Coniocarpon cinnabarinum* 2	*Frisch* (TRH-L-29000)	Norway	**MN733980**	**MN734115**	**MN719393**
*Coniocarpon cinnabarinum* 3	*Frisch* (TRH-L-29002)	Norway	**MN733982**	**MN734117**	**MN719395**
*Coniocarpon cinnabarinum* 4	*Johnsen 111003* (UPS)	Norway	KJ850976	–	KJ851103
*Coniocarpon cinnabarinum* 5	*Frisch* (TRH-L-29008)	Norway	**MN733984**	**MN734119**	**MN719397**
*Coniocarpon cinnabarinum* 6	*Frisch* (TRH-L-29001)	Norway	**MN733981**	**MN734116**	**MN719394**
*Coniocarpon cinnabarinum* 7	*Frisch 11/Ug297* (UPS)	Uganda	KJ850977	–	KJ851104
*Coniocarpon cinnabarinum* 8	*Frisch 11/Ug296* (UPS)	Uganda	KP870158	–	KP870170
*Coniocarpon cinnabarinum* 9	*Ertz 8730* (BR)	Rwanda	EU704046	–	EU704009
*Coniocarpon cinnabarinum* 10	*Frisch 13/Jp128* (TNS)	Japan	MG201840	–	–
*Coniocarpon cinnabarinum* 11	*Frisch 13/Jp127* (TNS)	Japan	MG201841	–	–
*Coniocarpon cuspidans* 1	*Frisch* (TRH-L-29026)	Norway	**MN733977**	**MN734113**	**MN719390**
*Coniocarpon cuspidans* 2	*Acton*, *Malíček*, *Palice 25146* (PRA)	Great Britain	**MN733979**	–	**MN719392**
*Coniocarpon cuspidans* 3	*Frisch* (TRH-L-29013)	Norway	**MN733970**	**MN734106**	**MN719384**
*Coniocarpon cuspidans* 4	*Frisch* (TRH-L-29025)	Norway	**MN733976**	**MN734112**	**MN719389**
*Coniocarpon cuspidans* 5	*Frisch* (TRH-L-29014)	Norway	**MN733971**	**MN734107**	**MN719385**
*Coniocarpon cuspidans* 6	*Klepsland* (TRH-L-29017)	Norway	**MN733973**	**MN734109**	**MN719387**
*Coniocarpon cuspidans* 7	*Frisch* (TRH-L-29022)	Norway	**MN733974**	**MN734110**	**MN719388**
*Coniocarpon cuspidans* 8	*Frisch* (TRH-L-29015)	Norway	**MN733972**	**MN734108**	**MN719386**
*Coniocarpon cuspidans* 9	*Frisch* (TRH-L-29023)	Norway	**MN733975**	**MN734111**	–
*Coniocarpon cuspidans* 10	*Frisch* (TRH-L-29024)	Norway	**MN733978**	**MN734114**	**MN719391**
*Coniocarpon fallax* 1	*Wågström 111123* (UPS)	Norway	MG201842	–	MG201850
*Coniocarpon fallax* 2	*Frisch* (TRH-L-29030)	Norway	**MN733967**	**MN734103**	**MN719382**
*Coniocarpon fallax* 3	*Gaarder*, *Larsen* (TRH-L-16791)	Norway	**MN733961**	**MN734097**	**MN719376**
*Coniocarpon fallax* 4	*Frisch* (TRH-L-29037)	Norway	**MN733968**	**MN734104**	**MN719383**
*Coniocarpon fallax* 5	*Gaarder* (TRH-L-16790)	Norway	**MN733959**	**MN734095**	**MN719374**
*Coniocarpon fallax* 6	*Gaarder* (TRH-L-16792)	Norway	**MN733960**	**MN734096**	**MN719375**
*Coniocarpon fallax* 7	*Gaarder* (TRH-L-16789)	Norway	**MN733963**	**MN734099**	**MN719378**
*Coniocarpon fallax* 8	*Gaarder* (TRH-L-15366)	Norway	**MN733962**	**MN734098**	**MN719377**
*Coniocarpon fallax* 9	*Frisch* (TRH-L-29029)	Norway	**MN733966**	**MN734102**	**MN719381**
*Coniocarpon fallax* 10	L10175	Great Britain	KJ850979	–	KJ851101
*Coniocarpon fallax* 11	*Frisch* (TRH-L-29028)	Norway	**MN733965**	**MN734101**	**MN719380**
*Coniocarpon fallax* 12	*Frisch* (TRH-L-29036)	Norway	**MN733969**	**MN734105**	–
*Coniocarpon fallax* 13	*Frisch* (TRH-L-29027)	Norway	**MN733964**	**MN734100**	**MN719379**
*Reichlingia leopoldii*	*Ertz 13293* (BR)	Belgium	JF830773	–	HQ454722
*Reichlingia syncesioides*	*Frisch 11/Ug14* (UPS)	Uganda	KF707651	–	KF707656
*Reichlingia zwackhii*	*Thor 26800* (UPS)	Sweden	KF707652	–	KF707662
*Synarthonia aurantiacopruinsa*	*Van den Broeck 5764* (BR)	DR Congo	MH251874	–	MH271697
*Synarthonia inconspicua*	*Van den Broeck 6325* (BR)	Uganda	MH251880	–	MH271701
*Synarthonia muriformis*	*Ertz 19344* (BR)	Madagascar	MH251877	–	MH271699

### Phylogeny

**Figure 1.**
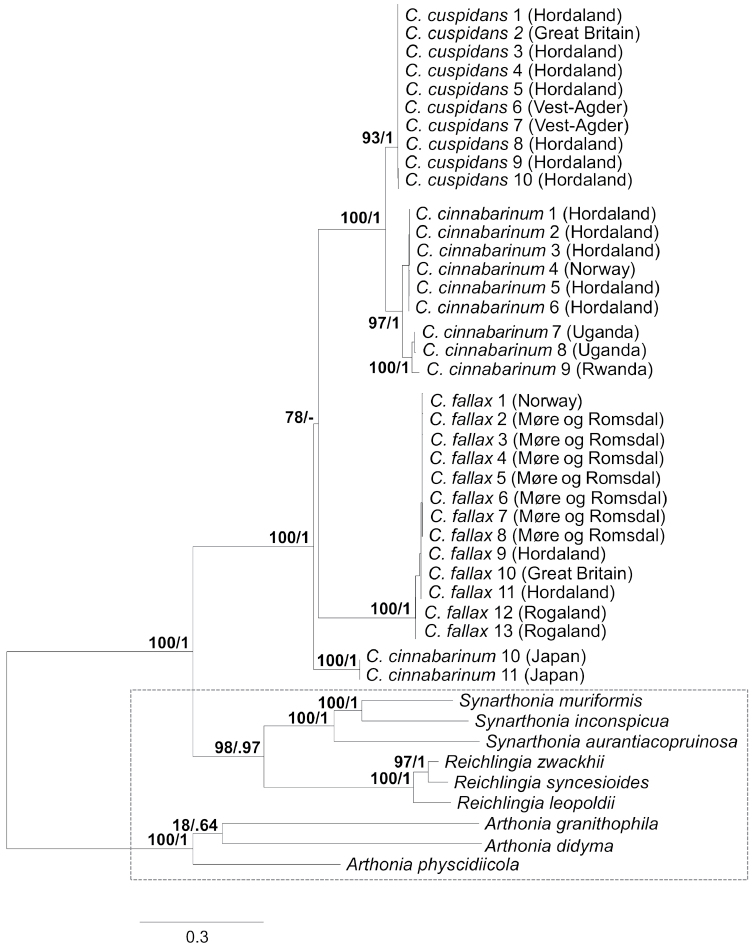
The RAxML phylogenetic hypothesis of a concatenated three-locus data set (mtSSU, nucITS and *RPB2*) of 3 *Coniocarpon* and 9 outgroup taxa (marked by a dashed rectangle). The RAxML bootstrap proportions (first) and Bayesian posterior probabilities (second) are indicated. Place of origin provided behind taxon names.

The phylogeny based on Bayesian and maximum likelihood analyses present *Coniocarpon* as monophyletic using the selected taxon and outgroup sampling. Three discrete, well-supported lineages are recovered within *Coniocarpon*. These are separated from each other by branches clearly exceeding the observed infraspecific branch-lengths. The three lineages represent *C. cinnabarinum*, *C. cuspidans* (Nyl.) Moen, Frisch and Grube and *C. fallax* (Fig. 1). *Coniocarpon cinnabarinum* is the supported sister taxon to *C. cuspidans*, while *C. fallax* is sister to these two taxa.

*Coniocarpon cinnabarinum* from Rwanda and Uganda form a well-supported clade and are sisters to *C. cinnabarinum* in Norway, while *C. cinnabarinum* from Japan is genetically distinct and sister to the remaining taxa of *Coniocarpon*. The two sampled specimens from Great Britain are genetically close to *C. cuspidans* and *C. fallax* in Norway, respectively.

### Morphology and chemical characters

**Figure 2.**
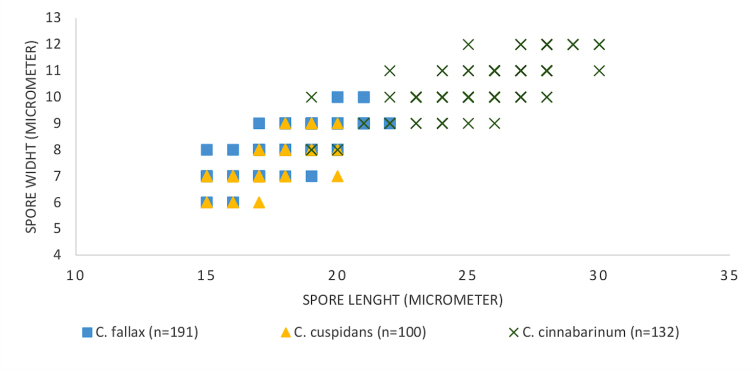
Ascospore size (length x width) of *C. cinnabarinum* (n = 132; 20 ascospores from Denmark, 85 ascospores from Norway; 27 ascospores from Sweden), *C. cuspidans* (n = 100, all ascospores from Norway) and *Coniocarpon fallax* (n = 191, all ascospores from Norway).

**Figure 3.**
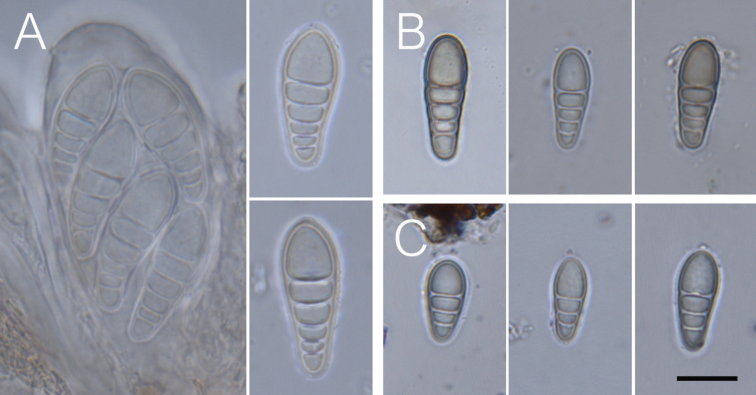
Ascospore morphology. **A***Coniocarpon cinnabarinum* (TRH-L-29007) **B***Coniocarpon fallax* (TRH-L-29008) **C***Coniocarpon cuspidans* (TRH-L-29036). Scale bar: 10 µm.

**Figure 4.**
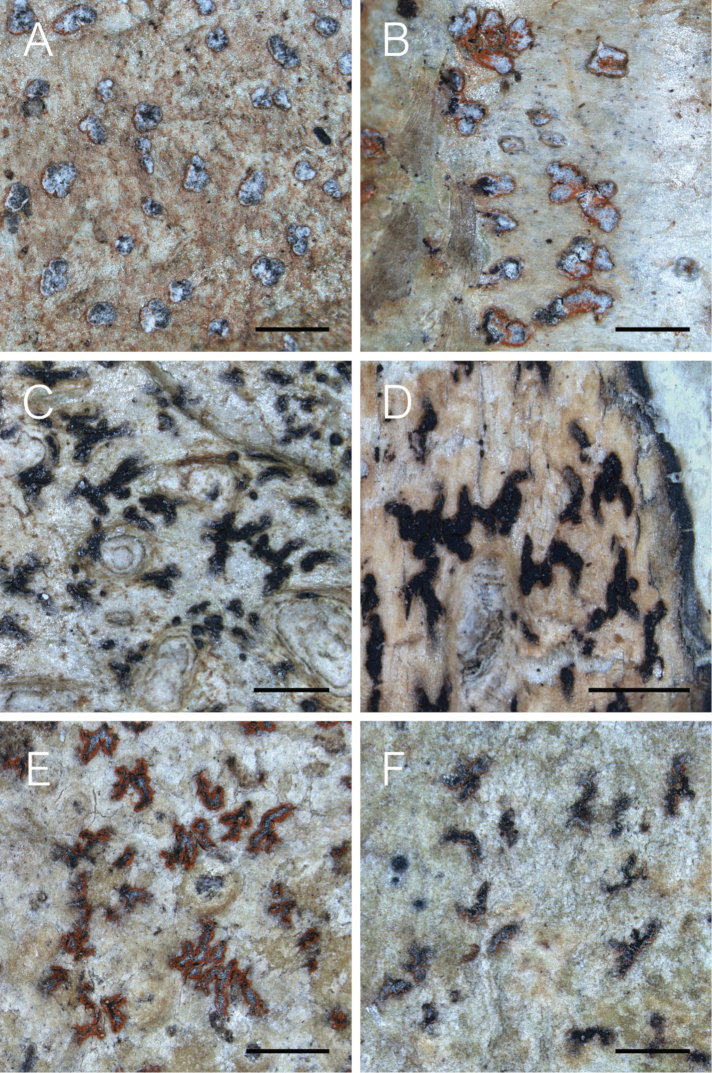
Morphological diversity of *Coniocarpon* in Norway. **A***Coniocarpon cinnabarinum* (*Frisch* TRH-L-29000) **B***Coniocarpon cinnabarinum* (*Frisch* TRH-L-29005) **C***Coniocarpon cuspidans* (TRH-L-29022) **D***Coniocarpon cuspidans* (TRH-L-29023) **E***Coniocarpon fallax* (TRH-L-16793) **F***Coniocarpon fallax* (*Frisch* TRH-L-29028). Scale bars: 1 mm.

**Figure 5.**
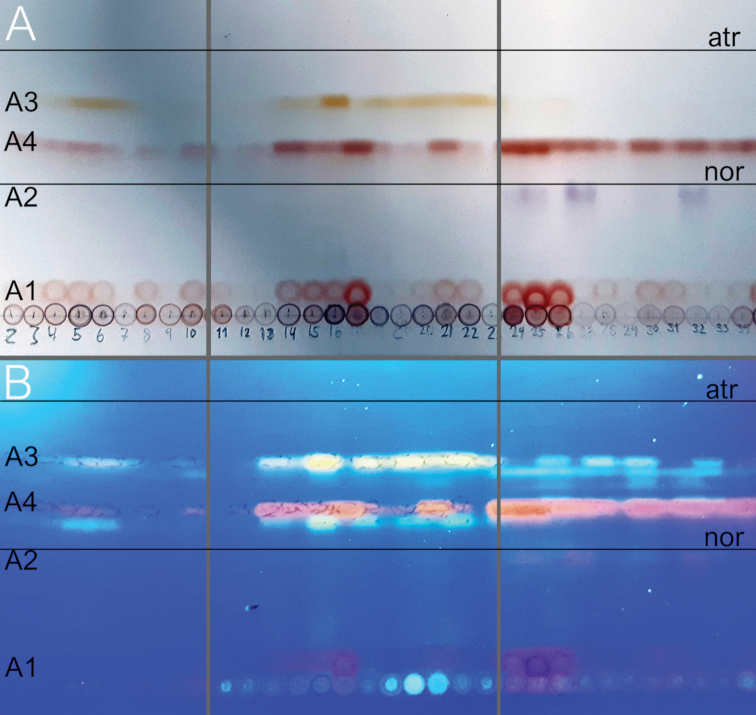
HPTLC plate in solvent C of quinoid pigment patterns of *C. cinnabarinum* (left), *C. fallax* (middle) and *C. cuspidans* (right) **A** before treatment with sulfuric acid and charring, and **B** under UV_365_ light before treatment with sulfuric acid and charring. Note the absence of A3 in *C. cuspidans*. The position of atranorin and norstictic acid is indicated. Several additional spots on the HPTLC plates under UV_365_ light have not been identified.

Forty-four specimens were identified as *C. cinnabarinum*, as *C. cuspidans* and 42 as *C. fallax*. Ascospore size (Fig. 2), ascospore septation (Fig. 3), ascoma shape and the distribution of pruina (Fig. 4) were identified as useful characters for species distinction. Moreover, differences were observed in the quinoid pigment patterns revealed by HPTLC (Fig. 5) and in the amyloidity of the ascomatal gels. Four quinoid pigments were identified showing different colors on the HPTLC plates prior to sulphuric acid treatment and charring; reddish (A1, A4), purple (A2), yellow (A3) (Fig. 5A). The color of the spots under UV_365_ light are deep purple (A1, A2), buff (A3) and dark salmon (A4) (Fig. 5B). The chemical results for all species are summarized in the Taxonomy section at the end of the Discussion.

### Distribution and ecology

**Figure 6.**
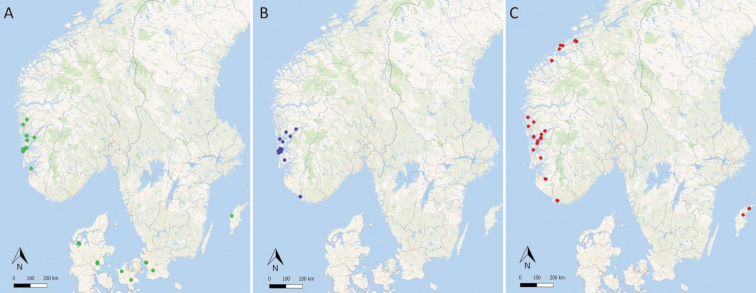
The distribution of 111 specimens of *Coniocarpon* in Norway, Sweden and Denmark based on material in BG, C, S, TRH and UPS. **A***C. cinnabarinum***B***C. cuspidans***C***C. fallax*. Single dots may represent several collections.

Distribution maps based on all revised specimens of *Coniocarpon* from Scandinavia confirm *C. cinnabarinum* for Denmark, Norway and Sweden, *C. cuspidans* for Norway, and *C. fallax* for Norway and Sweden (Fig. 6). *Coniocarpon cinnabarinum* has been collected in Norway in the boreo-nemoral rainforests in Rogaland and Hordaland, while the species occurs in other humid forests in Sweden (Skåne and Gotland) and Denmark (Sjælland and Jylland). Specimens of *C. cuspidans* have been seen in Norway from the boreo-nemoral rainforests in Vest-Agder, Rogaland and Hordaland. *Coniocarpon fallax* has been collected in Norway in the boreo-nemoral rainforests in Vest-Agder, Rogaland, Hordaland and Møre og Romsdal. This study further reports *C. fallax* from Sweden (Gotland) for the first time. Outside Scandinavia, *C. cuspidans* is confirmed for Great Britain and *C. fallax* for Austria, Great Britain, Switzerland and Turkey (not shown).

*Coniocarpon* preferably grows on trees with smooth bark and the selected host tree species slightly follow a geographical pattern (see Specimens examined below Taxonomic conclusions). Most collections of *C. cinnabarinum* from Norway have been made from *Corylus avellana* L., including few from *Fraxinus excelsior* L. and *Sorbus aucuparia* L. The species is collected in Denmark from *C. avellana*, *F. excelsior* and *Fagus sylvatica* L., and in Sweden from *F. excelsior*. Most specimens of *C. cuspidans* have been collected from *C. avellana*, but the species has been seen from a rather wide range of trees including *F. excelsior*, *Ilex aquifolium* L., *Quercus robur* L. and *S. aucuparia*. *Coniocarpon fallax* has mainly been collected from *F. excelsior* from Vest-Agder to Hordaland (more rarely from *C. avellana*), while all specimens from Møre og Romsdal are from *C. avellana*. The species is further collected from *F. excelsior* on Gotland and Austria, and from *Picea orientalis* (L.) Link in Turkey.

## Discussion

Species designations are hypotheses to be tested as new evidence becomes available. Recent molecular systematic studies have repeatedly revealed evolutionary lineages within phenotypically delimited lichenized fungi (e.g., Steinová et al. 2013; Lücking et al. 2014; Bendiksby et al. 2015; Alors et al. 2016; Hawksworth and Lücking 2017; Boluda et al. 2019). Whether or not such morphologically indistinguishable, or “cryptic”, evolutionary lineages should be recognized at species level may have critical implications for conservation biology and other fields of biology that use species as a fundamental unit (Lumbsch and Leavitt 2011).

In general, several factors should be considered in the process of assessing species status: proper selection of genetic markers (multiple, unlinked loci and from different genomic compartments), presence of statistically supported phylogenetic lineages, sufficiently large sample size, corroborating non-molecular character variation, and thorough review of the taxonomic and nomenclatural history (Grube and Kroken 2000; Printzen 2010; Lumbsch and Leavitt 2011). Our phylogenetic analyses of *Coniocarpon* are based on unlinked, multilocus DNA sequence data (mtSSU, nucITS, *RPB2*) showing high statistical support for three distinct genetic lineages (Fig. 1). Re-examination of morphology against the molecular phylogeny of 26 specimens revealed that the three lineages are further supported by differences in ascospore size (Fig. 2), ascospore septation (Fig. 3), distribution of pruina and ascoma shape (Fig. 4). Furthermore, the three lineages differ in the amyloidity of the ascomatal gels and pigment patterns revealed by HPTLC (Fig. 5). Finally, an additional 94 specimens, for which molecular data are not available, were revised using the same morphological and/or chemical characters. As such, this study fulfills the factors recommended for assessing species status.

Evolutionary lineages that remain intact when living in sympatry with close relatives might deserve species status (Coates et al. 2018). The distribution maps in Fig. 6 show sympatry for the three distinct genetic lineages of *Coniocarpon* in Norway, providing strong indirect evidence that there are mechanisms prohibiting exchange of genetic material among them, supporting their acceptance at species level. Hence, the integrated data gathered in this study jointly support the hypothesis of three distinct species of *Coniocarpon* in Norway, viz. *C. cinnabarinum*, *C. fallax* and *C. cuspidans*. The latter species was hidden in the extensive synonymy of *C. cinnabarinum* as Arthonia cinnabarina f. cuspidans Nyl. and is herein resurrected (see Taxonomic conclusions below).

The present data indicate a narrower distribution in Norway for *C. cinnabarinum* and *C. cuspidans* as compared to *C. fallax* (Fig. 6). All available collections from Møre og Romsdal were identified as *C. fallax*. *Coniocarpon cinnabarinum*, the only species reported from that county in the *Norwegian Red List of Species 2015* could not be confirmed. However, not all collections of *Coniocarpon* in Norway were available for this study, and previous distributions were partly based on human observations as well. The distribution of the three *Coniocarpon* species in Norway needs further evaluation in light of the present investigation. Moreover, this study confirms *C. cinnabarinum* for Denmark and Sweden. *Coniocarpon fallax* is reported from Sweden (Gotland) for the first time (Fig. 6), based on specimens from S and UPS previously identified as *C. cinnabarinum*.

Species diversity and abundance generally correlate with habitat preference. Most collections of *Coniocarpon* in Norway were made in Hordaland (*C. cinnabarinum* 22, *C. cuspidans* 24, *C. fallax* 20), in the core area of the boreo-nemoral rainforests, having the highest levels of humidity and low average winter temperatures (Blom et al. 2015; Moen 1999). In comparison, the number of studied specimens from Vest-Agder (*C. cinnabarinum* 0, *C. cuspidans* 2, *C. fallax* 3) at the southern limit of the boreo-nemoral rainforests is distinctly lower, which might be partly explained by fewer rainforest localities (Blom et al. 2015). The occurrence of *C. fallax* in Møre og Romsdal indicates a wider ecological amplitude as compared to *C. cinnabarinum* and *C. cuspidans* in terms of oceanity and temperature. *Coniocarpon cuspidans* is currently only confirmed for Norway and Great Britain, but as the species has not been distinguished from *C. fallax* in the past, it might have a wider distribution in Western Europe.

This study provides a further step in the process of improving the biodiversity conservation in Norway by applying an integrative taxonomic approach for delimiting taxa in *Coniocarpon*. *Coniocarpon cinnabarinum* and *C. fallax* are designated as VU in the latest version of the *Norwegian Red List for Species* (Henriksen and Hilmo 2015). It is likely that the same status applies to *C. cuspidans*, growing with similar abundance in the boreo-nemoral rainforests as the other species of *Coniocarpon*. Moreover, all three species are facing the same threats such as algae growth on trees which is inhibiting the establishment and growth of lichens (Blom 2018).

## Taxonomic conclusions

### *Coniocarpon* DC

In Lamarck and de Candolle, Flore française 2: 323 (1805) [MB 1208]. Lectotype (selected in Santesson, Symbolae Botanicae Upsalienses 12(1): 68, 1952): *Coniocarpon cinnabarinum* DC., in Lamarck and de Candolle, Flore française 2: 323 (1805).

#### Key to Coniocarpon in Norway

**Table:** 

1	Ascospores mostly > 20 µm long; ascomata typically rounded to weakly lobate, rarely lirellate; orange–red pruina present	***C. cinnabarinum***
–	Ascospores mostly ≤ 20 µm long; ascomata typically irregularly lirellate; orange–red pruina present or absent	**2**
2	Orange–red pruina present; ascospores (15–)17–20(–22) × (6–)7–9(–10) µm, (1–)3–4(–5) transversely septate	***C. fallax***
–	Orange–red pruina absent; ascospores (15–)16–18(–20) × (6–)7–8(–9) µm, (2–)3(–4) transversely septate	***C. cuspidans***

#### Coniocarpon cinnabarinum

Taxon classificationFungiArthonialesArthoniaceae

DC.

7E9519C4-EE2F-5FBF-BAAE-AEAE45C0AE10

383614

Figs 2 , 3A , 4A, B , 5 , 6A

Coniocarpon cinnabarinum DC.: Lamarck and de Candolle, Flore française 2: 323 (1805). Type: not selected [see under "Notes" below].
= Spiloma ? tumidulum Ach., Methodus qua omnes detectos lichenes: 11 (1803) [MB 405550]. Type: Hispania, Schousboe (H-Ach. 3 c!, holotype).
= Spiloma tumidulum var. rubrum Ach., Lichenographia universalis: 137 (1810) – nom. illegit. Type: Gallia (H-Ach. 2 c!).

##### Description.

Thallus pale olive gray to brown, weakly glossy to matt, smooth, endophloeodal to partly epiphloeodal, continuous; *prothallus line* dark gray to brown, sometimes present when in contact with other lichens; *photobiont* trentepohlioid, the cells rounded to elliptical, 7–12 × 4–8 μm, forming short chains. Ascomata irregularly rounded to elliptical to weakly lobed, rarely distinctly lirellate, with steep flanks, emergent from thallus, 0.1–0.4 × 0.1–0.3 mm, 95–140 µm tall, solitary or forming loose to dense aggregations of 3–15 ascomata, (0.3–)0.5–2.0(–3.5) × 0.3–1.6 mm; *disc* dark purple black, flat to weakly convex, matt to weakly glossy, white pruinose, a layer of orange–red pruina sometimes present above the white pruina; old ascomata sometimes epruinose; *margins* level with the disc, typically orange–red pruinose, sometimes with additional patches of white pruina; *proper exciple* brown, 8–15 µm wide, composed of compressed and vertically oriented paraphysoidal hyphae, the hyphae 1–2 µm thick, branched and netted, often forming short hairs up to 15 µm long at the outer margin; old bark cells often attached to the exciple; *epithecium* brown, 10–25 µm tall, conglutinated only in the lower parts, composed of branched tips of the paraphysoidal hyphae extending horizontally above the asci; the tips slightly widened to 3(–4) µm, sometimes extending from the epithecium as sparsely branched anticlinal hairs up to 22 µm long; *hymenium* hyaline, strongly conglutinated, (45–)65–90 µm tall, paraphysoids densely branched and netted, 1–2 µm thick; *hypothecium* hyaline, conglutinated, 20–35 µm tall, formed of irregular prosoplectenchymatic hyphae 1–2 µm diam.; *crystals* common in epithecium and proper exciple, of two types: hyaline, leafy crystals, 1–5(–8) µm, and orange, red or purple, granular crystals, 1–2(–4) µm; a weak amorphous, red to purple pigmentation present in exciple, epithecium and patchily distributed in the hymenium. ASCI of the *Arthonia*-type, long obpyriform to clavate, 62–84 µm × 24–35 µm (n = 34), 8-spored, the ascospores stacked; tholus 8–11 µm thick, lateral ascospore wall 1–2 µm thick. ASCOSPORES hyaline, (3–)4–5(–8) transversely septate, (19–)23–28(–30) × (8–)10–11(–12) µm (l: mean = 25.7, STD = 2.3; w: mean = 10.5, STD = 0.9; n = 132), obovate, with enlarged apical cell, getting pale brown with granular ornamentation in the epispore at late maturity; development macrocephalic.

##### Chemistry.

Pigments A1, A3 and A4 in variable amounts detected by HPTLC. *Proper exciple* I_dil_+ blue, I+ blue, KI+ blue; *epithecium* I_dil_+ blue, I+ blue, KI+ blue; *hymenium* I_dil_+ red, I+ red, KI+ blue; *hypothecium* I_dil_+ red, I+ red, KI+ blue. A hemiamyloid ring present in the tholus of the asci. Hyaline crystals dissolve in K. Orange, red and purple crystals dissolve in K with a clear, fleeting, purplish solution.

##### Specimens examined.

Norway – **Rogaland** • Rennesøy, Berge; 59°05.868'N, 05°42.320'E; on *C. avellana*; 30–40 m a.s.l.; 12. Jan. 2008; J. I. Johnsen leg.; BG L-86128. – **Hordaland** • Askøy, close to Ask farm; on *S. aucuparia*; 10–30 m a.s.l.; 31. Aug. 1909; J. J. Havaas leg.; UPS-L-277202. • Bømlo, Børøy, Storavatnet; 59°42.9420'N, 05°15.7680'E; on *C. avellana*; 30. Apr. 2018; G. Gaarder leg.; TRH-L-18030 • ibid.; Lykling; 59°42.6780'N, 05°12.3540'E; on *C. avellana*; 30. Apr. 2018; G. Gaarder leg.; TRH-L-18036 • ibid.; S of Liarnuten; on *C. avellana*; 21. Jun. 1997; T. Knutsson leg.; UPS-L-737333 • ibid.; Skogafjellet; 59°38.812'N, 05°12.098'E; on *C. avellana*; 35 m a.s.l., 19. Jul. 2017; A. Frisch leg.; TRH-L-29000 • ibid.; 59°38.818'N, 05°12.082'E; on *C. avellana*; 10 m a.s.l.; 19. Jul. 2017; A. Frisch leg.; TRH-L-29006, TRH-L-29007, TRH-L-29008 • ibid.; on *F. excelsior*; A. Frisch leg.; TRH-L-29009 • ibid.; 59°38.833'N, 05°12.153'E; on *C. avellana*; 50 m a.s.l.; 19. Jul. 2017; A. Frisch leg.; TRH-L-29001, TRH-L-29002. • Kvam, Gravdal SW, Geitaknottane Nat. Reserve, NE of Lønningshaugen; 60°06.690'N, 05°51.068'E; on *C. avellana*; 150–250 m a.s.l.; 28. Aug. 1997; P. G. Ihlen leg.; BG-L-35863. • Lindås, Kvalvika-Røyldalane; 60°38.338'N, 05°26.258'E; on *C. avellana*; 35 m a.s.l.; 14. May 2018; A. Frisch leg.; TRH-L-29010, TRH-L-29011, TRH-L-29012. • Os, Innerøya, Halhjem; 60°08.5020'N, 05°24.8520'E; on *S. aucuparia*; 10. May 2018; G. Gaarder leg.; TRH-L-18042 • ibid.; 60°08.5920'N, 05°25.3440'E; on *C. avellana*; 10. May 2018; G. Gaarder leg.; TRH-L-18043. • Stord, Digernes, Geitåsen; 59°45.402'N, 05°25.092'E; on *C. avellana*; 28. Apr. 2018; G. Gaarder leg.; TRH-L-18033 • ibid.; Valavåg, Nes-Åsen; 59°46.0740'N, 05°24.8040'E; on *C. avellana*; 27. Apr. 2018; G. Gaarder, U. Hanssen leg.; TRH-L-18087. • Tysnes, Beltestad, Beltestadknappen; 59°59.883'N, 05°27.543'E; on *C. avellana*; 13 m a.s.l.; 9. May 2018; A. Frisch leg.; TRH-L-29003, TRH-L-29004 • ibid.; 59°59.900'N, 05°27.555'E; on *C. avellana*; 5 m a.s.l.; 9. May 2018; A. Frisch leg.; TRH-L-29005. SWEDEN – **Gotland** • Stenkumla, Myrsö; 1869; Laurer leg.; UPS-L-002825. – **Skåne** • Dalby, Dalby Söderskog; on *F. excelsior*; 23. Jul. 1947; R. Santesson leg.; UPS-L-118296 • ibid.; Ottarp, Bälteberga; on *F. excelsior*; 20. Aug. 1946; O. Almborn leg.; S-F-71116, UPS-L-60625 • ibid.; 16. Sep. 1959; G. Degelius, O. Almborn leg.; UPS-L-60624. DENMARK – **Jylland** • Horsens, Elling Skov; on *F. excelsior*; 26. Mar. 1887; J. Jeppesen leg.; C-L-28996 • ibid.; on *F. sylvatica*; 26. Mar 1887; J. Jeppesen leg.; C-L-28993 • ibid.; on *F. excelsior*; 26. Feb. 1887; J. Jeppesen leg.; S-F-71202, C-L-28992 • ibid.; on *F. excelsior*; 20. Feb. 1887; J. P. Pedersen leg.; C-L-28991, C-L-28994 • ibid.; Hansted Skov; on *F. excelsior*; 5. Des. 1886; J. Jeppesen leg.; C-L-29000 • ibid.; 1. Feb. 1887; J. Jeppesen leg.; C-L-28999 • ibid.; on *F. sylvatica*; 6. Mar. 1887; J. Jeppesen leg.; C-L-28998. • Lihme, Kås skov; on *C. avellana*; 6. Aug. 1979; G. Thor leg.; UPS-L-165392 • ibid.; on *F. excelsior*; 25. May. 1976; S. Svane leg.; C-L-28997, C-L-28988 • ibid.; on *C. avellana*; 25. May 1976; M. S. Christansen leg.; C-L-28990 • ibid.; Bringsbjerg Krat; 56°37.129'N, 08°41.423'E; on *C. avellana*; 21. Oct. 2002; R. S. Larsen leg.; C-L-17076. – **Sjælland** • Haslev; 29. Jul. 1887; Taussieng leg.; C-L-28995 • ibid.; Skarresø; 4. Nov. 1870; C. Grönlund leg.; UPS-L-002896.

##### Notes.

*Coniocarpon cinnabarinum* differs from the other *Coniocarpon* species in Norway by distinctly larger ascospores and in ascospore septation: (19–)23–28(–30) × (8–)10–11(–12) µm, (3–)4–5(–8) transversely septate vs (15–)16–18(–20) × (6–)7–8(–9) µm, (2–)3(–4) transversely septate in *C. cuspidans* vs (15–)17–20(–22) × (6–)7–9(–10) µm, (1–)3–4(–5) transversely septate in *C. fallax*. Further, the ascomata in *C. cinnabarinum* are mostly irregularly rounded to elliptical and only rarely lirellate as in *C. cuspidans* and *C. fallax*. The ascomatal disc in *C. cinnabarinum* is typically covered by a thick layer of white pruina which may be overlaid by orange–red pruina, and the ascomatal margin is orange–red pruinose. In *C. cuspidans*, the ascomata completely lack orange-red pruina, while a thin white pruina may be occasionally present. In *C. fallax*, the distribution of pruina is similar to *C. cinnabarinum*, but the white pruina is less pronounced and may even be lacking. Additional differences have been observed in the reaction of proper exciple and epithecium to iodine: I_dil_/I+ blue in *C. cinnabarinum* and *C. fallax* vs I_dil_/I+ red in *C. cuspidans*. The quinoid pigments A1, A3 and A4 have been identified in *C. cinnabarinum* in various amounts. The quinoid patterns in *C. fallax* are similar, while in *C. cuspidans* A3 is absent or occurs in trace amounts only. The pigment A2 has only been found in *C. cuspidans*.

*Coniocarpon cinnabarinum* is the selected type species of *Coniocarpon* (Santesson 1952), but the name is preceded by *Spiloma tumidulum* (Acharius 1803) and possibly *Sphaeria gregaria*Weigel (1772). The situation is further complicated by the fact that the only specimen of *C. cinnabarinum* in PC that is unequivocally linked to the publication of *Flore française* and donated by de Candolle, represents *C. fallax*. Since *C. cinnabarinum* is a well established species and often cited in literature, we intend to propose this name for conservation. The typification of the species will be discussed in the proposal, which is currently under preparation.

#### Coniocarpon cuspidans

Taxon classificationFungiArthonialesArthoniaceae

(Nyl.) Moen, Frisch & Grube comb. nov.

3FE84EB8-9E53-56ED-8E40-6D30F922FECC

833812

Figs 2 , 3C , 4C, D , 5 , 6B

Arthonia cinnabarina f. cuspidans Nyl., Flora 59: 310 (1876) [MB 372360]. Type: Ilicicola in Hibernia, n. 6, Dough[ruagh Mountain], 1875, Larbalestier (H-Nyl 5607! lectotype, here selected).

##### Description.

Thallus pale brown to pale fawn to off white, matt to weakly glossy, smooth, endophloeodal to partially epiphloeodal, continuous; *prothallus line* dark gray to brown to black, sometimes present when in contact with other lichens; photobiont trentepohlioid, the cells rounded to elliptical, 6–13 × 5–11 µm forming short chains. Ascomata weakly elongate to irregularly lirellate, with steep flanks, emergent from thallus, 0.2–0.6 × 0.1–0.2 mm, 60–105 µm tall, typically forming loose to dense aggregations of 3–15 ascomata, weakly elongated to irregularly lirellate to stellate, 0.4–1.8(–2.5) × (0.1)0.3–1.0(–2.0) mm; *disc* black to dark purple black, flat to weakly convex, weakly glossy to matt, epruinose, rarely with patches of a thin white pruina; *margins* level with the disc, epruinose, rarely with patches of a thin white pruina; *proper exciple* brown, 7–20 µm wide, composed of compressed and vertically oriented paraphysoidal hyphae, the hyphae 1–2 µm thick, branched and netted, sometimes forming short hairs up to 16 µm long at the outer margin; old bark cells sometimes attached to the exciple; *epithecium* brown, 8–20 µm tall, conglutinated only in the lower parts, composed of branched tips of the paraphysoidal hyphae extending horizontally above the asci; the tips slightly widened to 3(–4) µm, sometimes extending from the epithecium as sparsely branched anticlinal hairs up to 12 µm long; *hymenium* hyaline, strongly conglutinated, 41–73 µm tall, paraphysoids densely branched and netted, 1–2 µm thick; *hypothecium* hyaline, conglutinated, 15–30 µm tall, formed of irregular prosoplectenchymatic hyphae 1–2 µm diam.; *crystals* common in epithecium and proper exciple, of two types: hyaline, leafy crystals, 1–5 µm, and red or purple granular crystals, 1–3 µm; a weak amorphous, red to purple pigmentation present in exciple, epithecium and patchily distributed in the hymenium. ASCI of the *Arthonia*-type, long obpyriform to clavate, 45–70 × 19–28 µm (n = 31), 8-spored, the ascospores stacked; tholus 4–8 µm thick, lateral ascospore wall 1–2 µm thick. ASCOSPORES hyaline, (2–)3(–4) transversely septate, (15–)16–18(–20) × (6–)7–8(–9) µm (l: mean = 17.4, STD = 1.2; w: mean = 7.5, STD = 0.7; n = 100), obovate, with enlarged apical cell, getting pale brown with granular ornamentation in the epispore at late maturity; development macrocephalic.

##### Chemistry.

Pigments A1, A2, A3 and A4 in variable amounts detected by HPTLC. *Proper exciple* I_dil_+ red, I+ red, KI+ blue; *epithecium* I_dil_+ red, I+ red, KI+ blue; *hymenium* I_dil_+ red, I+ red, KI+ blue; *hypothecium* I_dil_+ red, I+ red, KI+ blue. A hemiamyloid ring in the tholus of the asci not observed. Hyaline crystals dissolve in K. Purple crystals dissolve in K with hyaline solution. Red and purple crystals dissolve in K with purplish solution.

##### Specimens examined.

Norway – **Vest-Agder** • Flekkefjord, Hidra, Høgåsen; 58°13.585'N, 06°33.370'E; on *S. aucuparia*; 35 m a.s.l.; 15. Jul. 2017; J. Klepsland leg.; TRH-L-29017 • ibid.; Nonfjell; 58°13.445'N, 06°33.550'E; 5–25 m a.s.l.; 15. Jul. 2017; A. Frisch TRH-L-29022. – **Rogaland** • Tysvær, Svinali W; 59°25.098'N, 05°34.104'E; on *C. avellana*; 19. Oct. 2017; G. Gaarder leg.; TRH-L-18034. – **Hordaland** • Austevoll, Huftaøy, Bjelland farm NE; 60°05.000'N, 05°16.000'E; on *C. avellana*; 0–40 m a.s.l.; 6. Jun. 1996; T. Tønsberg leg.; BG-L-32077, BG-L-34115. • Bømlo, Børøy, Masterhaugane nord; 59°42.840'N, 05°15.5040'E; on *S. aucuparia*; 30. Apr. 2018; G. Gaarder leg.; TRH-L-18038, TRH-L-18078 • ibid.; 59°42.6420'N, 05°15.3540'E; on *C. avellana*; 30. Apr. 2018; G. Gaarder leg.; TRH-L-18040 • ibid.; Kuhillerdalen; 59°45.36'N, 05°16.82'E; on *C. avellana*; 70 m a.s.l.; 11. May 2015; J. B. Jordal, H. H. Blom leg.; TRH-L-16794 • ibid.; Lykling, Lyklingfjorden N; 59°42.321'N, 05°10.569'E; on *C. avellana*; 10–20 m a.s.l.; 13. May 1996; T. Tønsberg leg.; BG-L-31539 • ibid.; 59°42.300'N, 05°10.600'E; on *C. avellana*; 40–60 m a.s.l.; 1. Jun. 1997; S. Ekman leg.; BG-L-38200 • ibid.; Skogafjellet; 59°38.833'N, 05°12.153'E; on *C. avellana*; 50 m a.s.l; 19. Jul. 2017; A. Frisch TRH-L-29015 • ibid.; 59°38.812'N, 05°12.098'E; on *C. avellana*; 35 m a.s.l.; 19. Jul. 2017; A. Frisch leg.; TRH-L-29013, TRH-L-29014 • ibid.; 59°38.818'N, 05°12.082'E; on *C. avellana*; 10 m a.s.l.; 19. Jul. 2017; A. Frisch leg.; TRH-L-29023 • ibid.; on *S. Aucuparia*; 10 m a.s.l.; 19. Jul. 2017; A. Frisch leg.; TRH-L-29024 • ibid.; 59°38.972'N, 05°12.432'E; on *C. avellana*; 12 m a.s.l.; 19. Jul. 2017; A. Frisch leg.; TRH-L-29025, TRH-L-29026 • ibid.; S of Totlandstjørna; 59°41.172'N, 05°20.940'E; on *C. avellana*; 28. Jun. 2017; G. Gaarder leg.; TRH-L-18035 • ibid.; Totsida; 59°40.8180'N, 05°19.5540'E; on *I. aquifolium*; 27. Jun. 2017; G. Gaarder, M. Lorentzen leg.; TRH-L-18037 • ibid.; Våge; 59°43.6260'N, 05°13.5060'E; on *C. avellana*; 30. Apr. 2018; G. Gaarder leg.; TRH-L-18031. • Fusa, Holmefjord, Eikhaugen; 60°17.952'N, 05°39.867'E; on *Q. robur*; 30 m a.s.l.; 8. May 2018; A. Frisch leg.; TRH-L-29021. • Kvam, Nes N; 60°10.085'N, 05°55.535'E; on *F. excelsior*; 4. Jun. 2018; G. Gaarder leg.; TRH-L-18608. • Stord, Åsen SW of Sagvåg; 59°46.043'N, 05°24.593'E; on *C. avellana*; 50 m a.s.l.; 28. Apr. 2018; A. Frisch leg.; TRH-L-29019 • ibid.; 59°46.088'N, 05°24.757'E; on *C. avellana*; 33 m a.s.l.; 28. Apr. 2018; A. Frisch leg.; TRH-L-29020. • Sund, Steinsland; 60°12.199'N, 05°04.929'E; on *C. avellana*; 20–40 m a.s.l.; 9. Mar. 1997; T. Tønsberg leg.; BG-L-34117. • Tysnes, Beltestad, Beltestadknappen; 59°59.883'N, 05°27.543'E; on *C. avellana*; 13 m a.s.l.; 9. May 2018; A. Frisch leg.; TRH-L-29018.

##### Additional examined specimens.

Great Britain – **Scotland** • Argyll, Appin, Glen Stockdale; 56°34.383'N, 05°21.583'W; on *C. avellana*; 65–90 m a.s.l.; 5. Jun. 2018; A. Acton, J. Malíček, Z. Palice leg. 25146; PRA • ibid.; Westerness Locli Surnart, Reripole Ravine; on *Corylus* sp.; 10. Mar. 1983; B. J. Coppins leg.; BG-L-58163 • ibid.; Benderloch, Lochnell House; 17/88.38; on *Corylus*; 15–45 m a.s.l.; 4. Aug. 1980; B. J. Coppins leg. 8056; E • Mid Perth (VC 88), S side of Loch Earn, west of Ardvorlich cottage; 27/ 61.22(-3); 105–130 m a.s.l.; 8. Aug. 1980; B. J. Coppins & P. W. James leg.; E • Clyde Islands, Arran (VC 100), Glenashdale; 26/03.25; on *Corylus*; 4. Apr. 1984; B. J. Coppins leg. 10169; E • Westerness (VC 97), S side of Loch Sunart, Laudale Woods ravine 0.5 km W of Liddesdale; 17/77.59; 0–75 m a.s.l.; 9. Mar. 1983; B. J. Coppins & P. M. Jørgensen leg.; E • Devon, SW of Bridge Reeve; 21/65.13; on *Quercus petraea*; 1. Aug. 1972; P. Harrold leg.; E.

##### Notes.

*Coniocarpon cuspidans* is characterized by lirellate ascomata lacking orange–red pruina, while red and purple pigment crystals and a weak amorphous red to purple pigmentation is present in proper exciple and epithecium. The quinoid pigment A3 is absent or occurs in possible trace amounts only. A3 may correspond to orange pigment crystals observed in microscopical preparations of *C. cinnabarinum* and *C. fallax* but not in *C. cuspidans*. These crystals are located in the pruina of the ascomatal margin and disc. The ascospores in *C. cuspidans* are the smallest observed for the genus in Norway, (15–)16–18(–20) × (6–)7–8(–9) µm and (2–)3(–4) transversely septate. A hemiamyloid ring in the tholus of the asci, which is present in *C. cinnabarinum* and *C. fallax*, could not be observed in the investigated material. Further differences to *C. cinnabarinum* and *C. fallax* are discussed under those species.

The protologue of Arthonia cinnabarina f. cuspidans (Nylander 1876) cites specimens from Ireland and Cuba as original material: “Ilicicola in Hibernia (Larbalestier)” and “Exotica eadem datur in C. Wright. Cub. no. 123 a et b”. Nylander obviously considered the material from Ireland as the factual type collection. We have selected a specimen from the Nylander herbarium in Helsinki as the lectotype, which is the only specimen from Ireland that undoubtedly has been seen by Nylander. A possible syntype exists in BM: “Derryclare, Connemara, ilicicola, 1876 [BM000974345]”. Another specimen [BM000974347] from Larbalestier’s herbarium has a printed later label that only states “On young trees. Doughruagh Mountain and other places in Connemara”. The type status of this specimen is unclear. Both specimens have been seen by us as high-resolution pictures obtained from the data portal of BM.

#### Coniocarpon fallax

Taxon classificationFungiArthonialesArthoniaceae

(Ach.) Grube

0B26C80C-05ED-54D4-ABD7-EB485B7D6EB9

808766

Figs 2 , 3B , 4E, F , 5 , 6C

Coniocarpon fallax (Ach.) Grube: Frisch et al., Taxon 63: 737 (2014). Spiloma fallax Ach., Methodus qua omnes dectectos lichenes: 10 (1803) [MB 405518]. Type: Germania (H-Ach. 2 a!, lectotype, selected in Frisch et al., Taxon 63: 737, 2014).
= Spiloma elegans Ach., Lichenographia universalis: 135 (1810) [MB 405516]. Coniocarpon elegans (Ach.) Duby, Aug. Pyrami de Candolle Botanicon Gallicum: 675 (1830) [MB 383617]. Lichen elegans (Ach.) Lam. in Lamarck and Poiret, Encyclopédie méthodique, botanique, suppl. 3(1): 352 (1813) [MB 122540]. Arthonia elegans (Ach.) Almq., Kongliga Svenska vetenskaps-akademiens handlingar 17(6): 19 (1880) [MB 118959]. Type: Schleicher, Plantae Cryptogamicae Helvetiae exsiccatae, centuria5 no. 54 (S, lectotype, selected in Van den Broeck et al., Plant Ecology and Evolution 151: 346, 2018).

##### Description.

Thallus pale fawn to gray brown, weakly glossy to matt, smooth, endophloeodal to partly epiphloedal, continuous; *prothallus line* dark grey to brown, sometimes present when in contact with other lichens; *photobiont* trentepohlioid, the cells rounded to elliptical, 8–13 × 5–10 µm, forming short chains. Ascomata weakly elongate to irregularly lirellate, with steep flanks, emergent from thallus, 0.2–0.4 mm × 0.1–0.2 mm, 65–110 µm tall, typically forming loose to dense aggregations of 3–15 ascomata, weakly elongated to irregularly lirellate to stellate, 0.2–1.5(–2.3) × 0.1–1.8 mm; *disc* black to dark purple black, flat to weakly convex, weakly glossy to matt, epruinose or with a thin layer of white pruina; *margins* level with the disc, orange–red pruinose, sometimes with additional patches of white pruina; *proper exciple* brown, 7–20 µm wide, composed of compressed and vertically oriented paraphysoidal hyphae, the hyphae 1–2 µm thick, branched and netted, often forming short hairs up to 17 µm long at the outer margin; old bark cells sometimes attached to the exciple; *epithecium* brown, 10–20 µm tall, conglutinated only in the lower parts, composed of branched tips of the paraphysoidal hyphae extending horizontally above the asci; the tips slightly widened to 3(–4) µm, sometimes extending from the epithecium as sparsely branched anticlinal hairs up to 24 µm long; *hymenium* hyaline, strongly conglutinated, 35–70 µm tall, paraphysoids densely branched and netted, 1–2 µm thick; *hypothecium* hyaline, conglutinated, 15–30 µm tall, formed of irregular prosoplectenchymatic hyphae 1–2 µm diam.; *crystals* common in epithecium and proper exciple, of two types: hyaline, leafy crystals, 1–5(–7) µm, and orange, red or purple granular crystals, 1–2(–4) µm; a weak amorphous, red to purple pigmentation present in exciple, epithecium and patchily distributed in the hymenium. ASCI of the *Arthonia*-type, long obpyriform to clavate, 50–75 × 20–32 µm (n = 33), 8-spored, the ascospores stacked; tholus 5–8 µm thick, lateral ascospore wall 1–2 µm thick. Ascospores hyaline, (1–)3–4(–5) transversely septate, (15–)17–20(–22) × (6–)7–9(–10) µm (l: mean = 18.5, STD = 1.9; w: mean = 8.2, STD = 0.9; n = 191), obovate, with enlarged apical cell, getting pale brown with granular ornamentation in the epispore at late maturity; development macrocephalic.

##### Chemistry.

Pigments A1, A3 and A4 in variable amounts detected by HPTLC. *Proper exciple* I_dil_+ blue, I+ dark blue, KI+ dark blue; *epithecium* I_dil_+ blue, I+ dark blue, KI+ dark blue; *hymenium* I_dil_+ red, I+ red brown, KI+ dark blue; *hypothecium* I_dil_+ blue, I+ dark blue, KI+ dark blue. A hemiamyloid ring present in the tholus of the asci. Hyaline, crystals dissolve in K. Orange, red and purple crystals dissolve in K with purplish solution.

##### Specimens examined.

Norway – **Vest-Agder** • Lyngdalsfjord; on *F. excelsior*; 8. Apr. 1905; A. H. Magnusson leg.; S-F-71115 • ibid.; 1925; A. H. Magnusson leg.; S-F-71114 • ibid.; on *F. excelsior*; 25. Jan. 1939; A. H. Magnusson leg.; UPS-L-002899. – **Rogaland** • Gjesdal, Dirdal NE; on *F. excelsior*; 6. Oct. 1984; S. Hultengren leg.; UPS-L-654296 • ibid.; 58°49.810'N, 06°11.970'E; on *F. excelsior*; 160 m a.s.l.; 12. Jul. 2017; A. Frisch leg.; TRH-L-29036, TRH-L-29027. – **Hordaland** • Askøy; on *C. avellana* and *F. excelsior*; 1909; J. J. Havaas leg.; UPS-L-137313 • Fusa, Tveitane; 13 km S of Mundheim; 60°03'N, 05°52'E; on *C. avellana*; 150 m a.s.l.; 18. Aug. 1995; A. Nordin leg.; UPS-L-61739 • ibid.; Øvre Hålandsdalen, W of Orra; 60°15.5065'N, 05°55.137'E; on *F. excelsior*; 120 m a.s.l.; 24. Feb. 2015; S. Vatne leg.; TRH-L-16793. • Kvam, Daleelva N; 60°07.453'N, 05°52.721'E; on *F. excelsior*; 10. Jun. 2018; G. Gaarder leg.; TRH-L-18605 • ibid.; 60°07.668'N, 05°52.898'E; on *F. excelsior*; 13. Jun. 2018; G. Gaarder leg.; TRH-L-18606 • ibid.; Furhovda; 60°09.243'N, 05°53.910'E; on *F. excelsior*; 5. Jun. 2018; G. Gaarder, M. Lorentzen leg.; TRH-L-18604 • ibid.; Hovden; 60°13.735'N, 05°59.633'E; on *F. excelsior*; 11. Jun. 2018; G. Gaarder leg.; TRH-L-18607. • Lindås, Helltveit W; 60°37.872'N, 05°26.080'E; on *F. excelsior*; 24. Jul. 1980; T. Tønsberg leg.; BG-L-26222 • ibid.; Kvalvika-Røyldalane; 60°38.245'N, 05°26.345'E; on *F. excelsior*; 35 m a.s.l.; 14. May 2018; A. Frisch, J. Klepsland leg.; TRH-L-29035. • Os, Li; 60°10.38'N, 05°26.45'E; on *F. excelsior*; 60–120 m a.s.l.; 22. Jul. 1979; T. Tønsberg leg.; BG-L-26221, BG-L-26221. • Stord, Valavåg, Nes-Åsen; 59°46.116'N, 05°24.660'E; on *F. excelsior*; 27. Apr. 2018; G. Gaarder, U. Hanssen leg.; TRH-L-18041. • Tysnes, SE slope of Skardnipa near Teigen; 59°58.360'N, 05°39.008'E; on *F. excelsior*; 23 m a.s.l.; 9. May 2018; A. Frisch, J. Klepsland leg.; TRH-L-29031 • ibid.; 59°58.361'N, 05°39.023'E; on *F. excelsior*; 19 m a.s.l.; 9. May 2018; A. Frisch, J. Klepsland leg.; TRH-L-29032, TRH-L-29033 • ibid.; 59°58.350'N, 05°38.992'E; on *F. excelsior*; 24 m a.s.l.; 9. May 2018; A. Frisch, J. Klepsland leg.; TRH-L-29034 • ibid.; Sunde, Loksund; on F. excelsior; 27. Aug. 1910; J. J. Havaas leg.; UPS-L-137512. • Tysnes, Tysnesøy, N of Onarheim; 59°58.377'N, 05°39.047'E; on *F. excelsior*; 30 m a.s.l.; 21. Jul. 2017; A. Frisch leg.; TRH-L-29028, TRH-L-29029. – **Møre og Romsdal** • Fræna, S of Hustad, Lunheim; 62°55.3871'N, 07°06.892'E; on *C. avellana*; 60 m a.s.l.; 15. Apr. 2016; H. Holien leg.; TRH-L-17089 • ibid.; Nordmark E; 62°54.998'N, 07°07.068'E; on *C. avellana*; 80 m a.s.l.; 26. Apr. 1998; G. Gaarder leg.; BG-L-39619 • ibid.; Tverrfjell; 62°54.872'N, 07°16.243'E; on *C. avellana*; 60 m a.s.l.; 5. Jul. 2017; A. Frisch leg.; TRH-L-29037, TRH-L-29030. • Skodje, Igletjønna; 62°28.6565'N, 06°34.4845'E; on *C. avellana*; 20. Apr. 2014; G. Gaarder, P. Larsen leg.; TRH-L-16791. • Tingvoll, Kamsvågtrøa; 63°01.8998'N, 08°08.150'E; on *C. avellana*; 16. Feb. 2014; G. Gaarder leg.; TRH-L-15366 • ibid.; Langvatnet NE; 63°02.9460'N, 08°04.3020'E; on *C. avellana*; 8. Nov. 2014; G. Gaarder leg.; TRH-L-16792 • ibid.; Skjelberget; 63°03.2040'N, 08°03.8640'E; on *C. avellana*; 25. Apr. 2014; G. Gaarder leg.; TRH-L-16789 • ibid.; Årøyvatnet; 63°02.8320'N, 08°02.3940'E; on *C. avellana*; 8. Nov. 2014; G. Gaarder leg.; TRH-L-16790. SWEDEN – **Gotland** • Bunge, Hägur, Mörku, 1 km NE of the church; 57°51.00'N, 19°00.00'E; on *F. excelsior*; 15 m a.s.l.; 27. Apr. 1996; A. Nordin leg.; UPS-L-74225 • 600 m from Bunge church; on *F. excelsior*; 27. Apr 1996; G. Westling leg.; S L-52399; • Bäl, Gute, 1 km E Bäl church; 57°39.00'N, 18°40.00'E; on *F. excelsior*; 25. Nov. 1996; P. Johanson leg.; UPS-L-98398.

##### Additional examined specimens.

Austria – **Upper Austria** • Totes Gebirge, Lake Almsee SSE; 47°44.600'N, 13°57.400'E; on *F. excelsior*; 600 m a.s.l.; 31. May 1998; T. Tønsberg leg.; BG-L-66299. Turkey – **Trabazon** • Trabazon Vilayet, Uzungöl c. 14 km SSE of Caykara; 40°36.8700'N, 40°18.8500'E; on *P. orientalis*; 24. Jun. 2001; C. Printzen, B. Kanz leg.; BG-L-77481.

##### Notes.

*Coniocarpon fallax* resembles *C. cinnabarinum* by ascomata that are covered in an orange–red and white pruina. However, the white pruina is less pronounced in *C. fallax* and may be absent. The ascomata of *C. fallax* further are elongate to clearly lirellate and the ascospores are distinctly smaller with less septa: (15–)17–20(–22) × (6–)7–9(–10) µm, (1–)3–4(–5) transversely septate vs (19–)23–28(–30) × (8–)10–11(–12) µm, (3–)4–5(–8) transversely septate in *C. cinnabarinum*. Mature, apparently well-developed elliptical ascospores with only a single septum were found in one specimen (TRH-L-17089) of *C. fallax*. *Coniocarpon cuspidans* has ascospores of similar size which, however, are predominantly 3-septate. The elongate to lirellate ascomata of that species lack an orange–red pruina, and proper exciple and epithecium react I_dil_/I+ red in iodine.

## Supplementary Material

XML Treatment for Coniocarpon cinnabarinum

XML Treatment for Coniocarpon cuspidans

XML Treatment for Coniocarpon fallax
